# Characterization of therapeutic antibodies targeting lymphatic and vascular resistance factor for the treatment of hypervascularized and highly metastatic cancers

**DOI:** 10.1002/1878-0261.70343

**Published:** 2026-10-20

**Authors:** Marion David, Arthur Karaulic, Audrey Bennetot, Clémence Fournier, Quentin Odet, Arthur Gouraud, Isabelle Bourget, Cédric Gaggioli, Sandy Giuliano, Frédéric Luciano, Olivier Léger, Zaki Sellam, Gilles Pagès

**Affiliations:** ^1^ Kekkan Biologics Strasbourg France; ^2^ University Côte d'Azur, Institute for Research on Cancer and Aging of Nice (IRCAN) UMR CNRS 7284/U INSERM 1081 France; ^3^ University Côte d'Azur, Institute for Research on Cancer and Aging of Nice (IRCAN) UMR CNRS 7284/U INSERM 1081, 3D‐Hub‐S platform France

**Keywords:** Angio/lymphangiogenesis, metastasis, renal cancer, resistance treatment, therapeutic antibody

## Abstract

Angiogenesis is a major driver of tumor progression and a validated therapeutic target in highly vascularized cancers such as clear cell renal cell carcinoma. However, the clinical benefit of antivascular endothelial growth factor therapies is limited by the emergence of resistance. We identified a vascular endothelial growth factor A splice variant, termed Lymphatic and Vascular Resistance Factor (LVRF), which promotes both angiogenesis and lymphangiogenesis and is upregulated following anti‐angiogenic treatment. To therapeutically target this pathway, we generated fully human monoclonal antibodies against LVRF. Lead antibodies displayed high specificity and nanomolar affinity and effectively inhibited LVRF‐driven biological activities. Anti‐LVRF antibodies reduced renal carcinoma cell invasion, inhibited vascular and lymphatic endothelial responses, and suppressed lymphatic endothelial cell sprouting. *In vivo*, treatment significantly reduced the growth of bevacizumab‐resistant renal carcinoma xenografts, induced regression of blood and lymphatic vessels, and decreased metastatic dissemination in zebrafish models. These findings identify LVRF as a mediator of resistance to anti‐angiogenic therapy and support therapeutic targeting of LVRF as a novel strategy for vascularized tumors resistant to current antivascular endothelial growth factor treatments.

## Graphical abstract

LVRF (lymphatic and vascular resistance factor) is a VEGFA splice variant upregulated in response to anti‐angiogenic therapy that fosters tumor adaptation through enhanced vascularization, lymphangiogenesis, and metastatic dissemination. Targeting LVRF with fully human neutralizing antibodies disrupts these processes and limits resistant tumor growth, establishing LVRF as a clinically relevant therapeutic target.

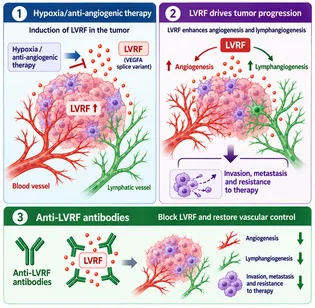

**Abbreviations**
BID: twice daily
ccRCC: clear cell renal cell carcinoma
CDR: complementarity‐determining region
CTLA4: cytotoxic T‐lymphocyte‐associated protein 4
EGF: epidermal growth factor
EGFR: epidermal growth factor receptor
HIF1/2α: hypoxia‐inducible factor 1/2 alpha
hpf: hours postfertilization
IP: intraperitoneal
KD: dissociation constant
KLH: keyhole limpet hemocyanin
LEC: lymphatic endothelial cell
LVRF: Lymphatic and Vascular Resistance Factor
LYVE‐1: lymphatic vessel endothelial hyaluronan receptor 1
MVD: microvascular density
OS: overall survival
OVA: ovalbumin
PD1: programmed cell death protein 1
PDL1: programmed death‐ligand 1
PDX: patient‐derived xenograft
PFS: progression‐free survival
PO: oral administration
PTU: 1‐phenyl‐2‐thiourea
scFv: single‐chain fragment variable
TGI: tumor growth inhibition
TKI: tyrosine kinase inhibitor
VEGFA: vascular endothelial growth factor A
VEGFC: vascular endothelial growth factor C
VEGFR1/2: vascular endothelial growth factor receptor 1/2
VH: variable heavy chain
VHL: Von Hippel–Lindau
VL: variable light chain

## Introduction

Tumors have a constant demand for nutrients and oxygen, as well as a mechanism to remove carbon dioxide and waste products. These crucial requirements are met through the process of angiogenesis, which leads to the development of tumor‐associated neovascularization. Throughout tumoral progression, angiogenesis and the resulting vascular network play a significant role in the metastatic spread of cancer cells, ultimately contributing to patient mortality [1, 2].

Angiogenesis is a finely balanced phenomenon, regulated by both pro‐angiogenic and anti‐angiogenic factors. In the context of cancer, this equilibrium is disrupted, with a shift toward an excess of pro‐angiogenic factors that promote aberrant neovascularization. The identification of vascular endothelial growth factor A (VEGFA) stands as a groundbreaking achievement in unraveling the mechanisms behind angiogenesis [3]. VEGFA plays a pivotal role in stimulating angiogenesis and enhancing vascular permeability by activating two critical tyrosine kinase receptors, VEGFR1/Flt1 (vascular endothelial growth factor receptor 1) and VEGFR2/KDR (vascular endothelial growth factor receptor 1/fms‐like tyrosine kinase 2/ kinase insert domain receptor.) [4]. The VEGFA/VEGFRs pathway holds a central position in driving the aggressiveness of clear cell renal cell carcinoma (ccRCC), which is the most prevalent subtype of renal cell carcinoma (RCC) [5]. In approximately 80% of ccRCC cases, the von Hippel–Lindau (VHL) tumor suppressor gene is inactivated, leading to the stabilization of hypoxia‐inducible factors 1 and 2 alpha (HIF1/2α) and the consequent overexpression of VEGFA [6].

In the case of nonmetastatic ccRCC patients, surgery stands as the established standard of care. Adjuvant therapy is typically considered for patients with evidence of local invasion. However, when the disease progresses to the metastatic stage, ccRCC proves to be unresponsive to conventional chemotherapy or radiotherapy. This refractory nature to standard treatments can be attributed to the tumor's hypervascularization [7, 8].

Given this unique context of hypervascularization, anti‐angiogenic therapies targeting VEGFA or its receptors (VEGFR1/2) have emerged as a promising treatment approach. Numerous clinical trials have confirmed the efficacy of these therapies in terms of prolonging progression‐free survival when compared to the previous reference treatment, interferon alpha. Subsequently, following the successful completion of these clinical trials, regulatory bodies such as the Food and Drug Administration and the European Medicine Agency approved the use of ATP mimetics like axitinib, sorafenib [9] and sunitinib [10] as a therapeutic option for metastatic ccRCC. In addition to sorafenib and sunitinib, the FDA (Food and Drug Administration) and EMA (European Medicines Agency) have also granted approval for bevacizumab, marketed under the brand name Avastin. Bevacizumab is an anti‐VEGFA monoclonal antibody, and it has been approved for the first‐line treatment of metastatic ccRCC. Importantly, bevacizumab is often used in combination with interferon alpha to enhance its therapeutic efficacy in treating metastatic ccRCC [11].

Given the important role that neovascularization plays in tumors, bevacizumab has also been approved for the treatment of metastatic colorectal cancer [12], nonsmall cell lung cancer [13], breast cancer [14], and ovarian cancer [15] in combination with standard chemotherapy for each of these cancers. Although the bevacizumab/chemotherapy combination prolonged progression‐free survival (PFS), its limited effect on overall survival (OS) led to the loss of FDA approval for breast cancer. Bevacizumab plus interferon lost FDA approval for renal cancer in 2016 but was recently approved in combination with the anti‐PD‐L1 (anti‐programmed death‐ligand 1) antibody atezolizumab as first‐line therapy for metastatic ccRCC [16]. The limited efficacy of bevacizumab and tyrosine kinase receptor inhibitors targeting angiogenesis, including VEGFR, can be attributed in part to the intricate biology of VEGFA. VEGFA is subject to regulation at multiple levels during its expression, encompassing transcription, pre‐mRNA splicing, mRNA stability, and translation [17]. One noteworthy aspect of VEGFA's complexity is its alternative splicing of pre‐mRNA, which results in the generation of mRNAs encoding various isoforms. The pro‐angiogenic isoforms are grouped as VEGFAxxx (e.g., VEGF121, VEGF165, VEGF189, and VEGF206, where XXX represents the number of amino acids minus the signal peptide of each isoform). In 2002, an alternative splice site in exon 8 of the human VEGF gene was identified, leading to the formation of the VEGFAxxxb family. VEGFAxxxb isoforms differ from VEGFAxxx by their last six amino acids (CDKPRR for VEGFAxxx and SLTRKD for VEGFAxxxb) [18]. While VEGFAxxx isoforms exhibit pro‐angiogenic, pro‐permeability, and pro‐migratory properties, VEGFAxxxb isoforms are believed to have a less potent effect on these parameters and are considered as anti‐angiogenic. The controversy surrounding VEGF‐Ax, resulting from continued translation beyond the stop codon to generate a longer VEGFA isoform containing the SLTRKD motif, adds further complexity to this field [19, 20]. The presence of natural inhibitors of VEGFA/VEGFR signaling, such as VEGFAxxxb isoforms, could potentially explain the limited efficacy of anti‐VEGFA/VEGFR therapies. Inhibiting these isoforms may inadvertently remove a brake on angiogenesis, ultimately promoting tumor development, as observed in the case of colon tumors [21]. However, it is crucial to note that the existence and significance of these VEGFA isoforms have remained highly controversial. Two contrasting theories persist regarding their existence. While some studies continue to report results on their biological roles in various pathophysiological conditions, others consider these isoforms to be experimental artifacts [22]. This ongoing debate highlights the intricate and evolving nature of our understanding of VEGFA biology and its potential impact on cancer and angiogenesis.

We have described several other mechanisms that produce resistance to anti‐VEGFA/VEGFRs, including lysosomal trapping of the tyrosine kinase inhibitor (TKI) sunitinib [23], decreased expression of the membrane associated phosphatase PTPRk (receptor‐type tyrosine‐protein phosphatase kappa) [24, 25] leading to EGF receptor activation and overexpression of ELR + CXCL (Glu‐Leu‐Arg + C‐X‐C Motif Chemokine Ligand) cytokines and their receptors.

We have also described that bevacizumab and sunitinib stimulate the production of VEGFC, an important driver of lymphangiogenesis [26]. Although both treatments reduced tumor volume, they favor the development of an alternative vessel network that promotes metastatic dissemination via lymphatic vessels. Therefore, all factors that stimulate the development of a lymphatic network favor relapse. Importantly, VEGFA also stimulates the production of immune checkpoints in T lymphocytes (programmed cell death protein 1/PD‐1, cytotoxic T‐lymphocyte‐associated protein 4/CTLA4, T‐cell immunoglobulin and mucin‐domain containing‐3/TIM‐3) [27]. Therefore, the pro‐angiogenic and pro‐immune cell depletion properties of VEGFA explain the current therapeutic standard in ccRCC, which combines anti‐angiogenics with immune checkpoint inhibitors.

Thanks to bioinformatic analysis of the sequence of the human VEGFA gene, the presence of a consensus splice acceptor site was visualized, located 21 bp upstream of the conventional AG splice acceptor site in the seventh intron. Using this acceptor site, the insertion of 23 bp creates a new open reading frame that allows translation in the domain thought to be the 3′‐untranslated region (3′UTR) of VEGFA mRNA. Thus, 64 additional amino acids are included in the forms of VEGFA resulting from this alternative splicing [28]. Theoretically, all previous splice variants of VEGFA could contain these additional bases. Some of them have been amplified by RT‐PCR in tumor samples, in particular the one corresponding to the most highly expressed form of VEGFA, VEGF165.

We have uncovered some significant and unexpected findings regarding this newly discovered form of VEGFA, now termed Lymphatic and Vascular Resistance Factor (LVRF). Notably, LVRF was found to stimulate both angiogenesis and lymphangiogenesis, and its expression was induced by the selection pressure of bevacizumab.

These findings have led to several key observations. LVRF is present at high levels in various cancer cell types, including kidney, lung, breast, and brain tumor cells. Its expression surpasses that of normal tissue. Tumors generated from cells with high LVRF expression grow faster and develop both blood and lymphatic vessel networks. Inhibiting LVRF in tumors generated from bevacizumab‐resistant cells impedes the growth of experimental renal cell carcinoma (RCC). Clinical data further support the relevance of this pathway. High levels of LVRF in the plasma of patients treated with the tyrosine kinase inhibitor sunitinib correlate with shorter progression‐free survival and overall survival. These compelling results underscore the significance of LVRF as a promising therapeutic target, particularly in highly vascularized RCC. Furthermore, these findings suggest the potential applicability of LVRF as a therapeutic target in other tumor types characterized by a high degree of vascularization.

Building on this proof of concept, we generated fully human anti‐LVRF monoclonal antibodies. The primary objective of our work is not only to demonstrate their antitumor efficacy but also to define the specific tumors for which their use could represent a significant therapeutic breakthrough. This research offers exciting possibilities for the development of novel and effective treatments for cancer patients with highly vascularized tumors, potentially leading to improved outcomes and survival.

## Materials and methods

### Analysis of LVRF expression in patient‐derived xenograft (PDX) and human tissues

With the Society Champions Oncology (https://www.championsoncology.com) (Cohort 1), we had access to 1246 patient‐derived xenografts from 1246 different tumors. RNA‐seq was performed on these different PDX and reads corresponding to VEGF isoforms containing the specific LVRF sequence 5′‐CCTTTGTTTTCCATTTCCCTCAG‐3′ were examined. Independent validation of LVRF expression was performed using TissueScan™ Cancer Survey cDNA arrays (OriGene Technologies, CSRT103), following the manufacturer's instructions. Quantitative PCR (qPCR) was conducted using primers designed to specifically amplify the human LVRF isoform. (LVRF‐F: CCTTTGTTTTCCATTTCCCT, LVRF‐R: TCTGTCGATGGTGATGGTGTG_169bp). Amplification reactions were performed under standard conditions, and relative expression levels were calculated using the 2^−ΔCt^ method (ΔCt = Ct_LVRF − Ct_housekeeping gene provided in the array).

### Cell culture

The 786‐O (RRID: CVCL_1051, ATCC) cells were cultured in high‐glucose DMEM supplemented with GlutaMAX™, pyruvate, and 5% fetal calf serum. 786‐O cells were purchased directly from the American Type Culture Collection (ATCC) in 2025, which performs STR profiling on all deposited lines prior to distribution as part of its authentication program. Sunitinib‐and axitinib‐resistant and treated 786‐O cells have been previously described [29] 786‐O cells were incubated under normoxic or hypoxic conditions for 24 h and 48 h in either 5% or 0% fetal calf serum with or without CoCl2 (100 μm, Sigma‐Aldrich). The lymphatic endothelial cells (LEC) (Promocell, C‐12216) were cultured in Microvascular endothelial cell growth medium (Pelobiotech). All experiments were performed using mycoplasma‐free cells.

### Quantitative real‐time PCR (qPCR)

Total RNA was extracted using the NucleoSpin RNA kit (Macherey‐Nagel) and reverse‐transcribed with the QuantiTect Reverse Transcription kit (QIAGEN). qPCR was performed using SYBR Green Master Mix (Eurogentec). LVRF expression was quantified using specific primers (Forward: 5′‐CCTTTGTTTTCCATTTCCCT‐3′; Reverse: 5′‐TCTGTCGATGGTGATGGTGTG‐3′) and normalized to 36B4 expression (Forward: 5′‐CAGATTGGCTACCCAACTGTT‐3′; Reverse: 5′‐GGCCAGGACTCGTTTGTACC‐3′). Relative LVRF expression was quantified using the 2^‐ΔΔCt method. The LVRF forward primer was designed to target the additional exon, enabling the selective amplification of the LVRF‐specific sequence generated by the alternative splicing event. Consequently, this primer does not recognize or amplify canonical VEGFA transcripts.

### ELISA binding assays

ELISA plates (Maxisorp™) were coated overnight with recombinant human or murine LVRF, VEGFA, or LVRF peptide (10 μg·mL^−1^). After blocking with 1% BSA, serial dilutions of monoclonal anti‐LVRF antibodies or bevacizumab were added for 1 h at 37 °C. Bound antibodies were detected using an horseradish peroxidase (HRP)‐conjugated anti‐human IgG secondary antibody (Thermo Fisher Scientific), followed by 3,3′,5,5′‐tetramethylbenzidine (TMB) substrate development. Absorbance was measured at 450 nm.

### Spheroid invasion

Tumor spheroids were generated by culturing cells in Dulbecco's modified Eagle medium (DMEM) supplemented with 10% fetal bovine serum (FBS). Cells were seeded in round‐bottom 96‐well plates (Cellstar®) and embedded after 48 h in a collagen I/Matrigel matrix (Corning). Anti‐LVRF or isotype control antibodies were added and renewed every two days. Spheroids were monitored until day 7 using an EVOS microscope. Cell viability was assessed using the CellTiter‐Glo® assay (Promega), and spheroid invasion area and grayscale intensity were quantified with ImageJ.

### Phage display screening procedure

Phage display screening was performed by ProteoGenix using a proprietary human naïve Fab library (Premium LiAb‐Fab). Briefly, biopanning (4–6 rounds) was conducted against the peptide C‐DVTSRGGEPGRRKEPPSGFREPDLS, which is specific to LVRF and absent from the VEGFA sequence. To minimize nonspecific interactions, the peptide was conjugated to different carrier proteins (KLH, BSA, and OVA), and depletion steps were performed against the carriers alone to eliminate nonspecific binders. Positive clones were identified by ELISA, validated against the full‐length protein, sequenced, and ranked based on specificity and binding activity. The five best candidates were synthesized, reformatted as human IgG1κ antibodies, expressed by GenScript, and further characterized by ELISA against both peptide and full‐length LVRF.

### Lymphatic endothelial cells (LECs) sprouting

LEC sprouting was assessed using a fibrin bead assay. Briefly, LECs were coated onto gelatin microcarrier beads and embedded in a fibrin matrix. Human fibroblasts were seeded on top of the gels, and cultures were stimulated with recombinant LVRF (200 ng·mL^−1^) in the presence or absence of anti‐LVRF monoclonal antibodies (100 μg·mL^−1^). After 6–7 days, sprouting was quantified by fluorescence microscopy as the number of sprout‐positive beads per well.

### *In vivo* tumor growth

Animal studies were conducted by Axis Bioservices Ltd (Coleraine, UK) in accordance with the UK Animals (Scientific Procedures) Act 1986 and European Directive 2010/63/EU, under Project License 2862 and Establishment License 5030 issued by the Department of Agriculture, Environment and Rural Affairs (DAERA), Northern Ireland. Study protocols were approved by the Axis Bioservices Animal Welfare and Ethical Review Body (AWERB). Female Nu/Nu mice (5–8 weeks, 18–22 g; Janvier Laboratories) were acclimatized for 7 days and housed in individually ventilated cages with environmental enrichment, with free access to food and water under controlled temperature (18–24 °C), humidity (55–70%), and a 12‐h light/dark cycle.

786‐O ccRCC cells (1 × 10^7^) were implanted subcutaneously. Once tumors reached 20–100 mm^3^, mice were randomized into treatment groups based on tumor volume to ensure balanced baseline tumor sizes across groups. In experiment 1, mice (*n* = 5–8/group) received vehicle or axitinib (10 mg·kg^−1^, per os [PO], twice daily). In experiment 2, mice (*n* = 7/group) received isotype control, P1F12, P2H3, or P1B7 (7.5 mg·kg^−1^, intraperitoneally [IP], twice weekly). Tumor volume, body weight, and clinical signs were monitored throughout the study. Humane endpoints included tumor volume > 1500 mm^3^, tumor ulceration, > 20% body‐weight loss, or persistent signs of pain or distress. At study completion, animals were euthanized using a rising concentration of CO_2_ in accordance with Schedule 1 of the Animals (Scientific Procedures) Act 1986.

### Immunohistochemistry (IHC) analysis of tumor tissues

Tumor samples from the 786‐O xenograft study (Axis Bio) were formalin‐fixed, paraffin‐embedded, and analyzed by immunohistochemistry using anti‐Ki67 (Cell Signaling Technology), anti‐CD31 (Cell Signaling Technology), and anti‐LYVE‐1 (lymphatic vessel endothelial hyaluronan receptor 1) (Cell Signaling Technology) antibodies to assess proliferation, blood, and lymphatic vessel density, respectively. Whole‐slide digital image analysis was performed using QuPath (v0.5). Representative regions from each staining were used to train a computational pathology classifier following color deconvolution. Blood and lymphatic vessels were automatically detected and morphometrically classified. Collapsed vascular structures were defined using a computational pathology approach based on quantitative morphometric features extracted from CD31‐ and LYVE‐1‐positive vessels, including luminal area, circularity, thickness (calculated as area/[perimeter/2]), and perimeter. Vessels exhibiting a markedly reduced luminal area, together with decreased circularity and thickness and an increased perimeter, were classified as collapsed vessels.

### Zebrafish xenograft model and antibody treatment

Vybrant™ DiI‐labeled 786‐O cells were injected into the perivitelline cavity of 48‐h postfertilization (hpf) zebrafish larvae. Successfully xenografted larvae were treated with anti‐LVRF P2H3 or control antibody (250 μg·mL^−1^) and maintained at 33 °C in E3 embryo medium supplemented with 1× PTU (1‐phenyl‐2‐thiourea). Metastatic dissemination was assessed 72‐h postinjection by fluorescence microscopy through quantification of distant metastatic foci.

### Statistical analysis

All analyses were performed in GraphPad Prism. Statistical significance was determined by one‐way Tukey ANOVA, Dunnett's ANOVA, or Mann–Whitney test. A two‐sided *P*‐value of 0.05 or less was considered significant. Data are presented as the mean ± standard error of the mean (SEM).

## Results

### LVRF is broadly expressed across human solid tumors and enriched in aggressive, advanced stage, and metastatic disease

To systematically evaluate the clinical relevance of LVRF, we first evaluated its mRNA expression using two independent and complementary datasets. This approach allowed us to validate LVRF's expression patterns across a broad spectrum of human solid tumors while minimizing potential biases associated with individual cohorts. In Cohort 1, 1246 patient‐derived xenograft (PDX) models from Champions Oncology were analyzed for LVRF transcript reads. LVRF expression was detected in 62 PDX models (4.98%), with the highest frequencies observed in uterine (16.7%, 1/6 tumors), kidney (14.8%, 4/27 tumors), prostate (13.3%, 2/15 tumors), ovarian (12.1%, 7/58 tumors), pancreatic (10.4%, 8/77 tumors), and breast (10.1%, 12/119) (Fig. 1A). These tumor types are known for their high vascular demand and propensity for metastasis, suggesting a potential link between LVRF expression and tumor aggressiveness. Pathological characterization of these 62 LVRF‐positive tumors revealed a strong association with clinically aggressive disease. Specifically, 74% were poorly differentiated, 71% were classified as stage III–IV, and 58% were derived from metastatic lesions (Fig. 1B). These findings strongly suggest that LVRF expression is not only associated with tumor presence but also correlates with advanced disease stages and metastatic progression, which are key drivers of poor clinical outcomes. To further validate these findings, LVRF mRNA expression was quantified by qPCR in Cohort 2, comprising 379 samples across 12 cancer types, along with normal human tissues from TissueScan™ cDNA arrays. LVRF expression was detected in approximately 50% of normal tissues, likely reflecting its role in physiological angiogenesis, and ~70% of tumor samples. In most cases, LVRF was upregulated in tumors relative to matched normal tissues (Fig. 1C). The most pronounced tumor‐to‐normal increases were observed in prostate (21‐fold), breast (fourfold), ovarian (fourfold), lung (twofold), and kidney cancers (twofold). These independent datasets identify LVRF enriched in advanced and biologically aggressive tumors. Notably, several tumor types with elevated LVRF expression, including kidney, ovarian, lung, and breast cancers, are commonly treated with bevacizumab or related anti‐VEGFA strategies as part of standard clinical management. This convergence between LVRF expression and tumor contexts reliant on anti‐angiogenic therapy suggests that LVRF may participate in adaptive responses to VEGFA blockade and supports further investigation of LVRF as a potential therapeutic vulnerability and biomarker of treatment resistance. While LVRF may play a role in physiologic angiogenesis in normal tissues, its upregulation in tumors likely reflects pathological activation in response to hypoxia, metabolic stress, or therapeutic pressure.

**Fig. 1:**
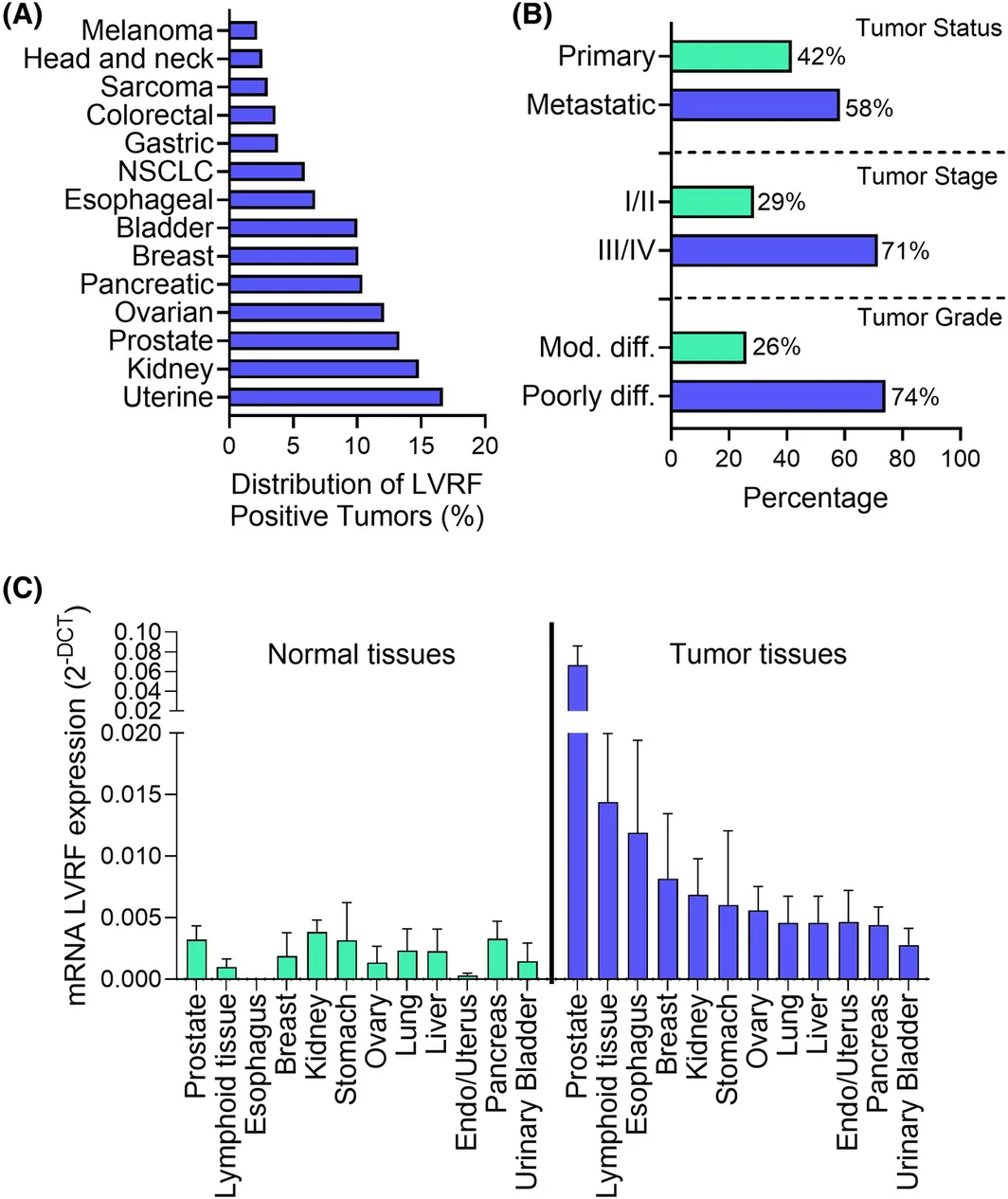
Lymphatic and vascular resistance factor (LVRF) is widely expressed in solid tumors, with a preference for aggressive, high grade, and metastatic disease contexts. (A) Percentage of LVRF‐positive patient‐derived xenograft (PDX) within each cancer type (Cohort 1, *n* = 1246, RNA‐seq analysis on PDX models). (B) Clinical and pathological characteristics of LVRF‐positive PDX models (Cohort 1), illustrating the distribution of tumor status (primary vs. metastatic), disease stage (I/II–III/IV), and histological tumor grade (moderately vs. poorly differentiated) across all 62 LVRF‐positive models, represented as percentages of the total. (C) Average relative LVRF mRNA expression levels (RT‐qPCR, 2^−ΔCT^) in normal (left) and tumor (right) tissue specimens from 12 tissue types using the TissueScan™ cDNA arrays (Cohort 2, *n* = 379 samples). Data are presented as mean ± SEM.

### LVRF expression is induced by hypoxia and upregulated upon TKI treatment in renal cell carcinoma

LVRF expression is linked with poor differentiation, advanced stage, and metastasis. Therefore, we next investigated whether stress factors within the tumor microenvironment may influence its expression. We first focused on hypoxia, a well‐known driver of VEGFA through HIF1/2α‐dependent pathway.

Using 786‐O RCC cells, we show that hypoxia (1% O_2_) induces LVRF mRNA expression, with a synergistic increase observed when hypoxia is combined with FBS starvation (Fig. 2A). Pharmacological stabilization of HIF2α (786‐Ocells only express HIF2α) with cobalt chloride (CoCl_2_) under normoxic conditions recapitulates this effect (Fig. 2B), supporting the notion that HIF2α can acts as a regulator of LVRF transcription. Given that anti‐angiogenic, receptor tyrosine kinase inhibitors (RTKIs) such as sunitinib and axitinib generate vascular stress and adaptive hypoxia in renal tumors, we next assessed their impact on LVRF expression. Acute treatment of 786‐O cells with sunitinib or axitinib resulted in a marked upregulation of LVRF mRNA within 48 h, reaching 5.2‐fold and 6.6‐fold increases, respectively. Notably, cell lines with acquired resistance to these TKIs maintained constitutively elevated LVRF expression, compared to parental controls, with increases of 2.9‐fold and 1.8‐fold, respectively (Fig. 2C).

**Fig. 2:**
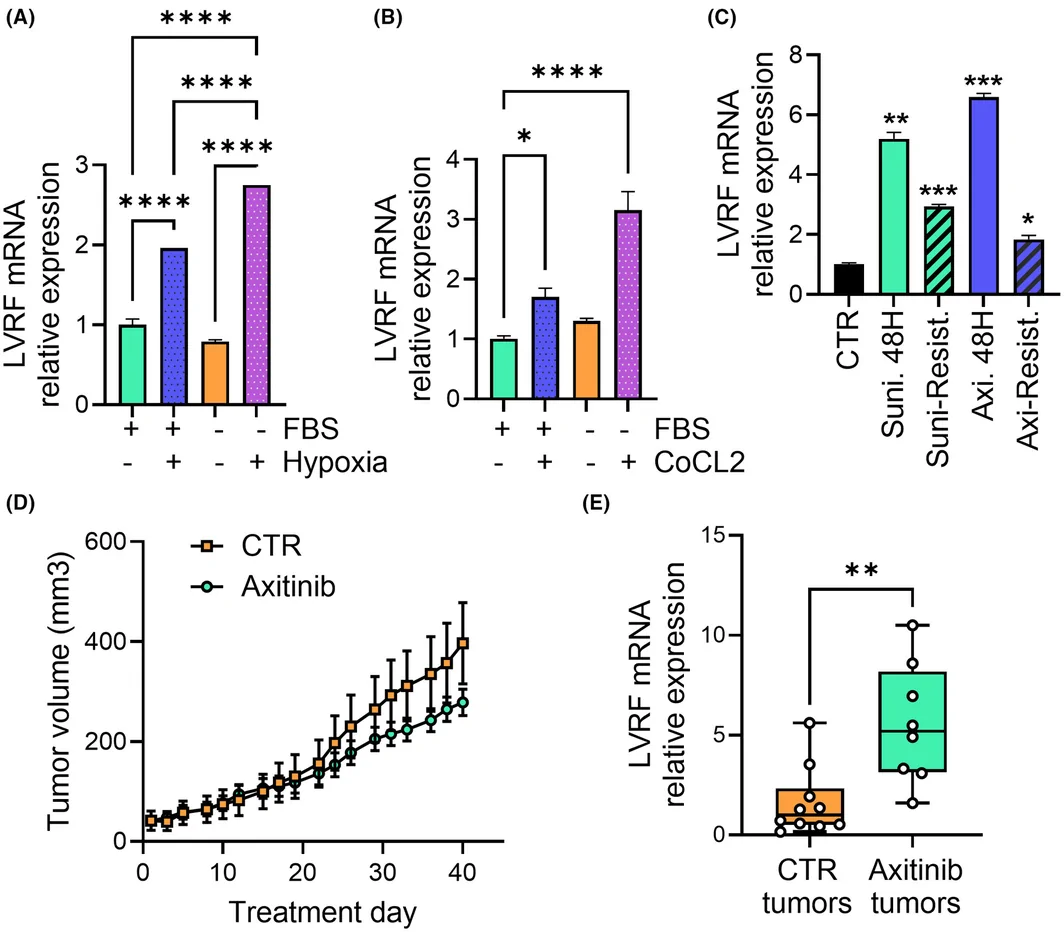
LVRF expression is induced by hypoxic stress and upregulated in response to tyrosine kinase inhibitor (TKI) treatment *in vitro* and *in vivo*. LVRF mRNA relative expression was quantified by RT‐qPCR in 786‐O renal cell carcinoma cells exposed to hypoxia (1% O_2_) and/or serum starvation for 48 h (A), treated with cobalt chloride (CoCl_2_) and/or serum starvation for 24 h (B). LVRF mRNA was determined in 786‐O cells following a unique treatment to sunitinib or axitinib (48H), and in sunitinib‐ or axitinib‐resistant derivative cell lines generated by prolonged drug exposure (C). (D) Tumor growth curves of 786‐O xenografts in mice treated with axitinib (*n* = 8) or vehicle control (CTR) (*n* = 5) over 40 days. (E) LVRF mRNA relative expression in tumors from axitinib‐treated and control mice (day 40). Data are presented as mean ± SEM or as box‐and‐whisker plots with individual data points (E). *n* = 3 independent experiments (A–C). Statistical significance was determined by one‐way ANOVA with Tukey's multiple comparisons test (A, B), one‐way ANOVA with Dunnett's T3 multiple comparisons test (C) or Mann–Whitney *U*‐test (E). *P < 0.05, **P < 0.01, ***P < 0.001, ****P < 0.0001.

While both RTKIs induced LVRF expression, the magnitude of upregulation in resistant cells was modestly higher in Sunitinib‐resistant models. This discrepancy may reflect the distinct cellular adaptations driven by Sunitinib's lysosomotropic properties, which are absent in Axitinib. As previously reported [23, 30], Sunitinib's sequestration in lysosomes disrupts autophagic flux and promotes inflammatory signaling, potentially contributing to a more pronounced stress response. However, our recent comparative transcriptomic and proteomic analysis of Sunitinib‐ and Axitinib‐resistant cells [29] revealed that, despite some differences in gene and protein expression profiles, LVRF upregulation remains consistent across both resistance models. This suggests that its induction is primarily driven by shared adaptive mechanisms, such as hypoxia and nutrient deprivation, rather than drug‐specific effects.

To validate this observation *in vivo*, 786‐O xenograft tumors were established in mice and treated with axitinib for 40 days. Despite continuous treatment, tumors exhibited progressive growth (Fig. 2D). RT‐qPCR analysis at endpoint revealed significantly higher LVRF mRNA levels in axitinib‐treated tumors compared to controls (3.4‐fold increase; Fig. 2E), confirming that TKI‐driven induction of LVRF occurs in a physiologically relevant tumor context. Collectively, these results establish LVRF as a stress‐responsive factor regulated by the convergence of hypoxic signaling and anti‐angiogenic pressure, which may also induce nutrient deprivation conditions analogous to serum starvation *in vitro*. While these results were derived from the highly relevant 786‐O ccRCC model, which recapitulates key features of anti‐angiogenic resistance, further validation across a broader panel of tumor models will be essential to define the full spectrum of LVRF‐dependent cancers. This integrated stress response highlights LVRF as a key adaptive mechanism in tumor cells and provides a strong rationale for the development of targeted anti‐LVRF therapeutic antibodies.

### Generation and characterization of anti‐LVRF antibodies via phage display screening

In this study, we employed a phage display strategy to generate anti‐LVRF antibodies using a peptide antigen corresponding to the LVRF‐specific sequence, as detailed in the Materials and Methods section. Following an initial round of bio‐panning, 96 positive clones were identified. Among these, 16 unique sequences were found to bind the peptide, and 12 of them also recognized the full‐length LVRF protein, supporting their selection for recombinant antibody production and further characterization (Fig. S1).

The corresponding sequences were used to generate recombinant human IgG1 antibodies, which were subsequently evaluated by ELISA for their binding to both the peptide and full‐length LVRF protein. Ten antibodies exhibited detectable binding, among which four clones, P1B7, P2H3, P1F12, and P1C9, demonstrated the strongest reactivity against both human and murine full‐length LVRF as well as the LVRF/NF peptide (Fig. 3A–C). Binding curves showed rapid saturation at low antibody concentrations, consistent with high‐affinity interactions.

**Fig. 3:**
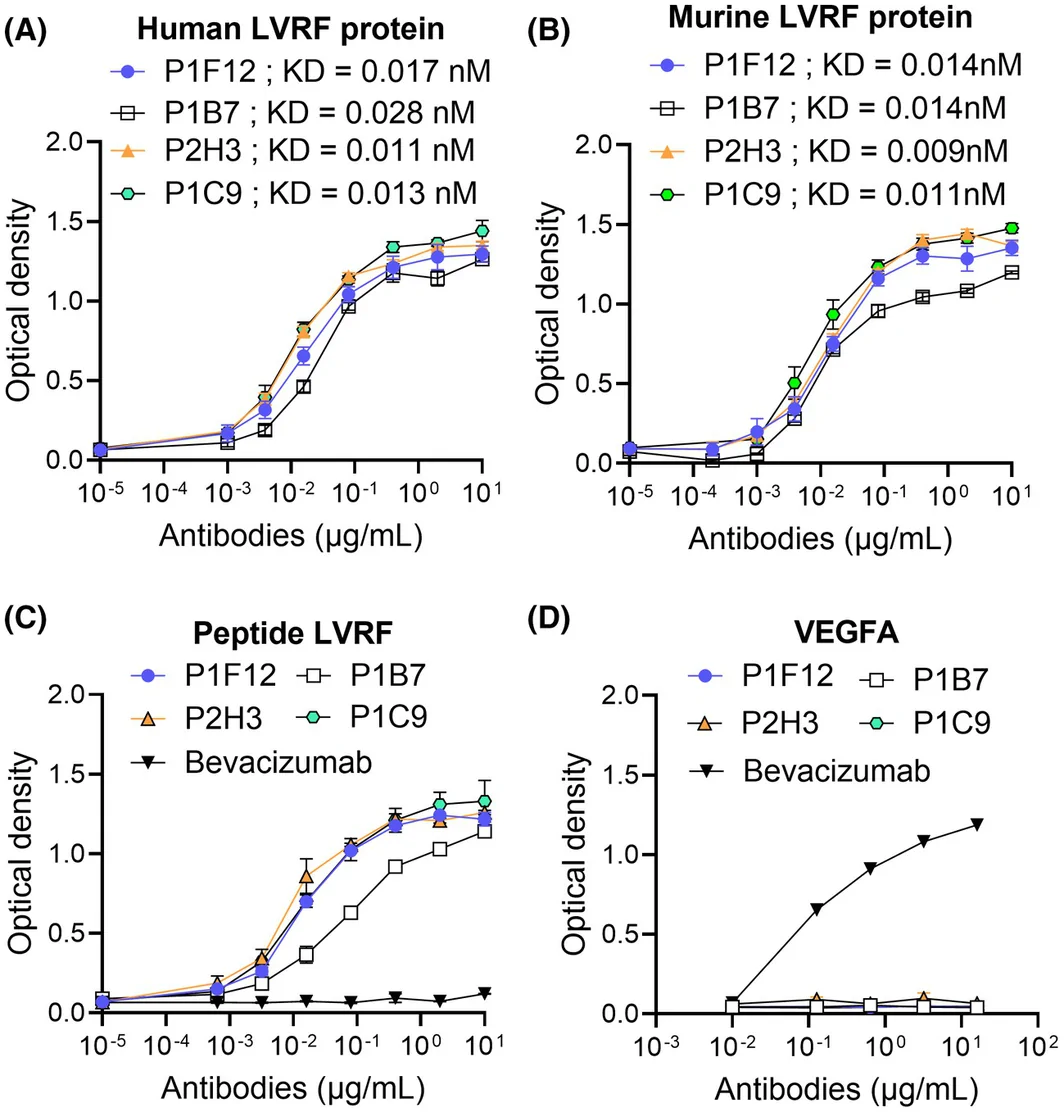
Generation and characterization of monoclonal anti‐LVRF antibodies via phage display. ELISA binding curves for the four selected antibodies: P1B7, P2H3, P1F12, and P1C9 against human LVRF protein (A) (*n* = 12–14), murine LVRF protein (B) (*n* = 6–10), LVRF peptide (NF domain) (C) (*n* = 2–4), and VEGFA (vascular endothelial growth factor A) protein (D) (*n* = 2). Bevacizumab is included as a comparator in panels C and D. Apparent dissociation constant (KD) values were estimated from ELISA binding curves A and B. Data are presented as mean ± SEM.

Quantitative analysis revealed subnanomolar dissociation constants (KD) for all four antibodies against both human and murine LVRF proteins. Specifically, KD values were 0.017 nm and 0.014 nm for P1F12, 0.028 nm and 0.014 nm for P1B7, 0.011 nm and 0.009 nm for P2H3, and 0.013 nm and 0.011 nm for P1C9, respectively (Fig. 3A,B). Importantly, all four antibodies selectively recognized the LVRF‐specific NF‐domain peptide and the full‐length LVRF protein, but did not bind VEGFA, demonstrating high specificity for the LVRF C‐terminal sequence without cross‐reactivity to canonical VEGFA family members (Fig. 3C,D). In contrast, bevacizumab bound shared VEGFA/LVRF regions but failed to recognize the LVRF‐specific NF domain.

The variable heavy (VH) and light (VL) chain sequences and the specific complementarity‐determining regions (CDRs) of the four selected antibodies are presented in Fig. 4. These results establish the successful generation of highly specific, high‐affinity anti‐LVRF antibodies with strong translational potential.

**Fig. 4:**
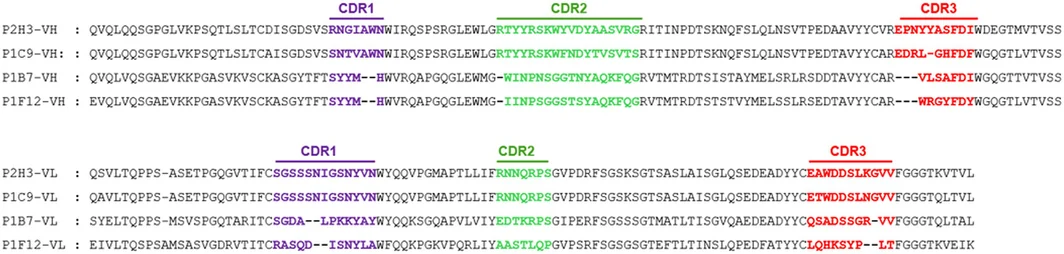
Variable domain sequences of anti‐LVRF antibodies. The figure displays the protein sequences of antibodies, showcasing both the heavy chains variable (VH) and the light chains (VL) regions. Complementary‐determining regions (CDR1, CDR2 and CDR3) are highlighted. The antibody sequences were displayed manually one below another to facilitate visual comparison.

### Anti‐LVRF antibodies suppress lymphangiogenesis and reduce tumor spheroid invasiveness and viability *in vitro*

To investigate the functional impact of LVRF neutralization on vascular remodeling and tumor aggressiveness, anti‐LVRF antibodies were evaluated using complementary lymphangiogenesis and 3D tumor spheroid models (Fig. 5). In a bead sprouting assay performed with primary human lymphatic endothelial cells (LECs) embedded in fibrin gels and supported by fibroblasts, recombinant LVRF significantly increased the number of sprout‐forming LEC‐coated beads compared with unstimulated controls, confirming its pro‐lymphangiogenic activity [28, 31]. Treatment with anti‐LVRF antibodies, including P2H3, P1B7, and P1C9, markedly inhibited LVRF‐induced sprouting and restored responses to near‐basal levels, demonstrating an efficient blockade of LVRF‐driven lymphangiogenesis (Fig. 5A and Fig. S2).

**Fig. 5:**
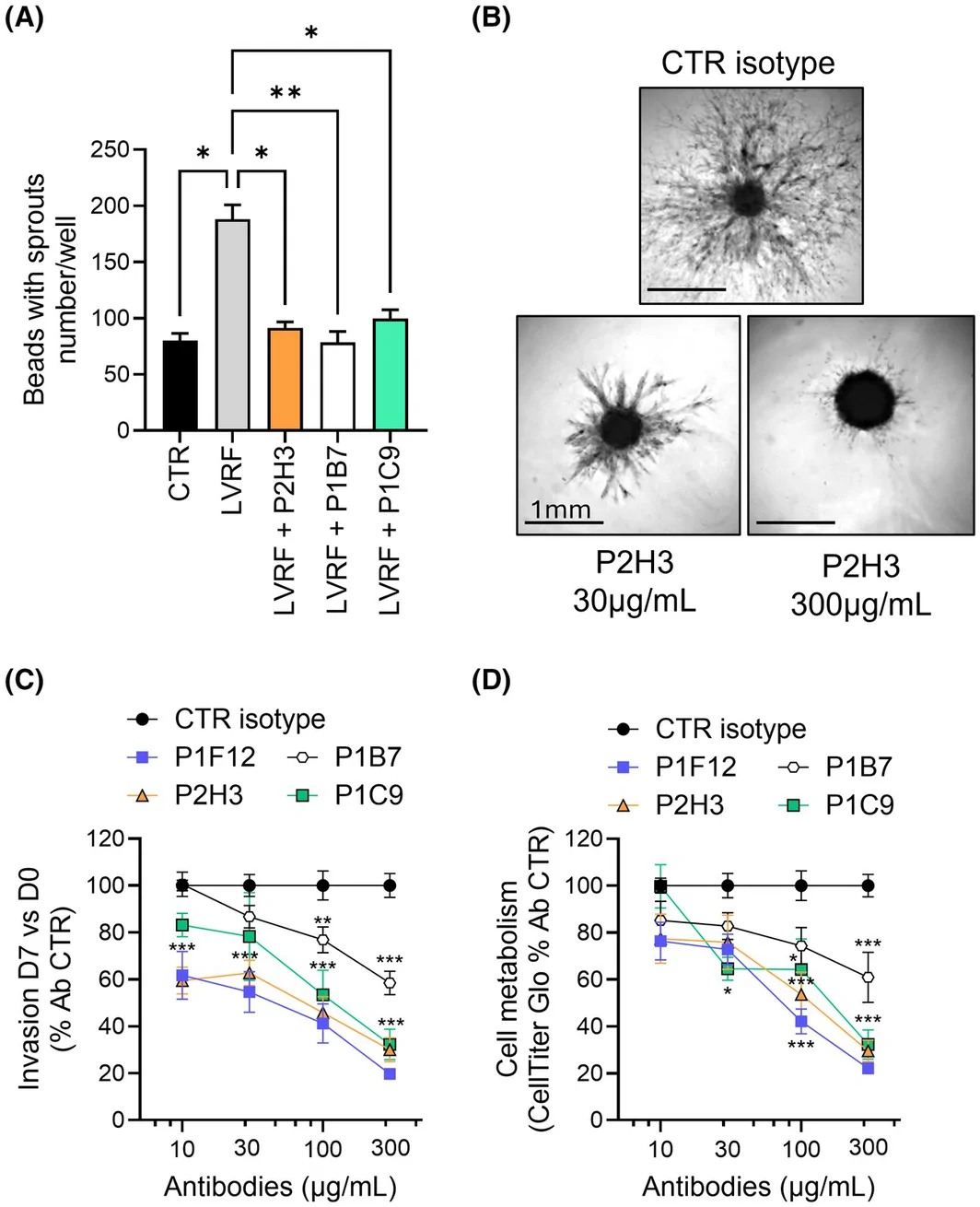
Anti‐LVRF antibodies inhibit lymphangiogenesis, decrease tumor spheroid invasion and metabolism. (A) Quantification of lymphatic endothelial cell (LEC) sprouting in a 3D bead assay under control conditions, stimulation with recombinant LVRF (200 ng·mL^−1^), or LVRF in the presence of anti‐LVRF antibodies (100 μg·mL^−1^) (*n* = 3). (B) Representative images of 786‐O clear cell renal cell carcinoma (ccRCC) spheroids cultured in a 3D invasion assay under control conditions or treated with antibodies (30 or 300 μg·mL^−1^), scale bars, 1 mm. Quantification of spheroid invasion area (C) (*n* = 4–18), and cell metabolism activity measured by CellTiter‐Glo assay, expressed as percentage of control following treatment with increasing concentrations of antibodies (10–300 μg·mL^−1^) (D) (*n* = 4–18). Data represent mean ± SEM and were analyzed by one‐way ANOVA with Dunnett's T3 multiple comparisons test. *P < 0.05, **P < 0.01, ***P < 0.001.

We next assessed whether LVRF neutralization also affects tumor cell behavior using 3D spheroids generated from 786‐O cells. Representative images obtained with the P2H3 antibody revealed a clear reduction in spheroid growth and invasive protrusions following treatment (Fig. 5B). Quantitative analyses confirmed that the hit antibodies significantly decreased cancer cell invasion in a 3D collagen, laminin‐rich matrix in a dose‐dependent manner (Fig. 5C). This inhibitory effect was accompanied by a reduction in cellular metabolic activity, as measured by CellTiter‐Glo directly on the spheroids (Fig. 5D).

Collectively, these results demonstrate that LVRF neutralization simultaneously impairs lymphangiogenic signaling and reduces tumor invasive capacity associated with a decrease in cell viability, thereby reinforcing the therapeutic relevance of targeting LVRF in cancer.

### Anti‐LVRF antibodies inhibit tumor growth *in vivo* in a bevacizumab‐resistant ccRCC model and impair tumor vascularization

To evaluate the *in vivo* therapeutic potential of anti‐LVRF antibodies, we used a xenograft model of bevacizumab‐resistant ccRCC. 786‐O cells were subcutaneously implanted in nude mice, and once tumors reached the exponential growth phase, animals were treated with anti‐LVRF antibodies at 7.5 mg·kg^−1^ administered intraperitoneally twice weekly for 40 days. All three anti‐LVRF antibodies tested significantly inhibited tumor growth compared to the isotype control (Fig. 6A). P2H3 induced complete tumor regression in 43% of cases and achieved a tumor growth inhibition (TGI) of 58%, while P1B7 led to 57% complete regression with a similar TGI of 58%. Notably, P1F12 also induced 43% complete regression but exhibited the strongest overall efficacy, with a TGI of 64%.

**Fig. 6:**
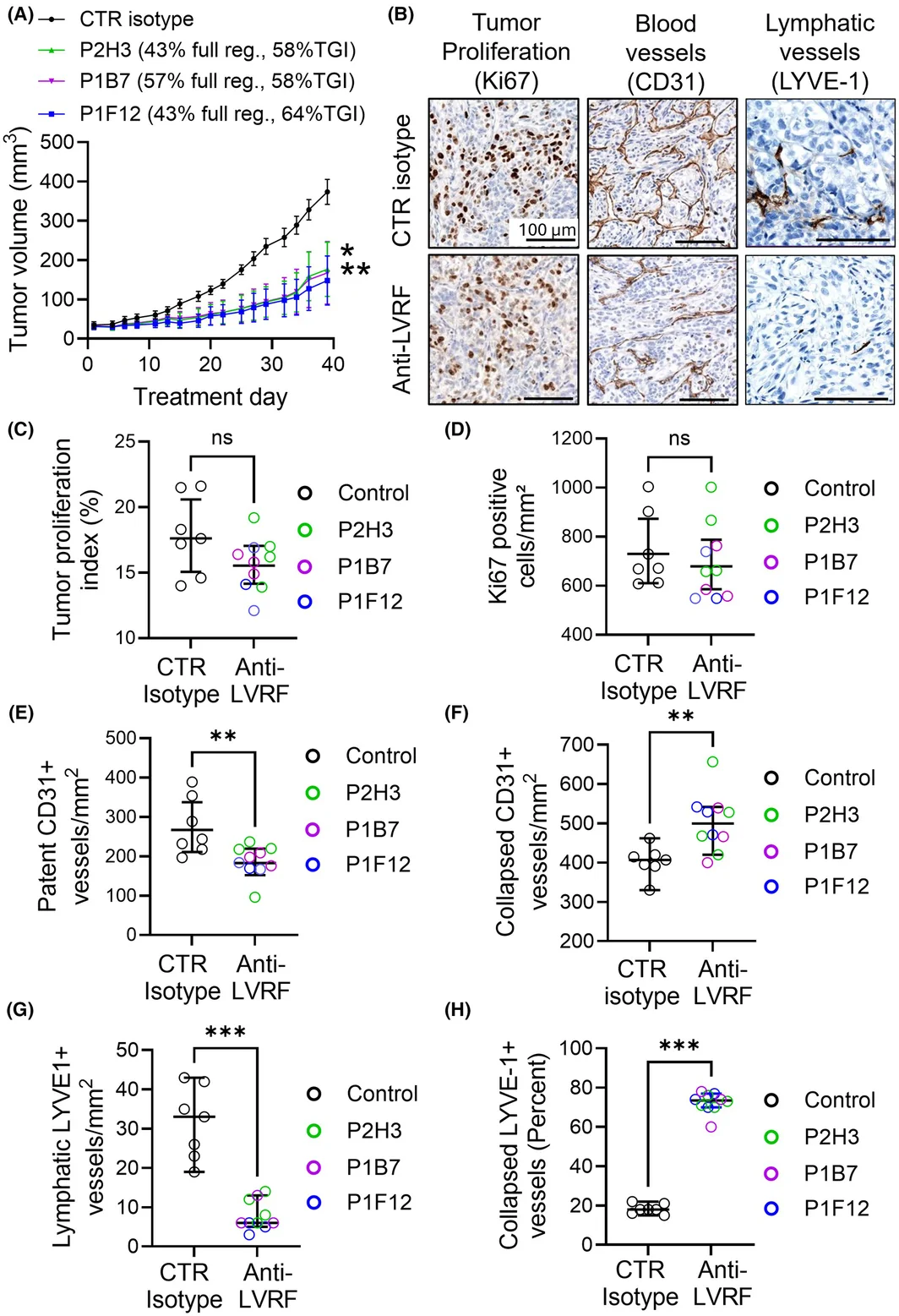
*In vivo* efficacy of anti‐LVRF antibodies against bevacizumab‐resistant ccRCC. (A) Tumor volume over 40 days of treatment in nude mice bearing 786‐O xenografts. Mice were treated with anti‐LVRF antibodies (P2H3, P1B7 and P1F12; 7.5 mg/kg intraperitoneally twice by week) or isotype control antibodies (*n* = 7/group). Tumor growth inhibition (TGI) and complete regression rates are indicated. (B) Representative immunohistochemical staining of tumor sections for cell proliferation (Ki67, left panels) and blood vessels (CD31, median panels) and lymphatic vessels (lymphatic vessel endothelial hyaluronan receptor 1: LYVE‐1, right panels) in control versus anti‐LVRF‐treated tumors. Scale bars, 100 μm. Quantification of Ki67 labelling (C) (Proliferation Index = number of Ki67+ tumor cells/Total number of tumor cells *100), and (D) density of Ki67+ cell per mm^2^. Patent per mm^2^ (E) and percentage of collapsed (F) CD31+ vessels by IHC (*n* = 7–10). Quantification of LYVE‐1+ lymphatic vessel density per mm^2^ (G) and percentage of collapsed lymphatic vessels by IHC (H) (*n* = 7–10). Data are presented as mean ± SEM in panel A. For panels C–H, individual data points are shown; horizontal lines indicate the geometric mean, and error bars represent the 95% confidence interval (95% CI). Statistical analysis: Mann–Whitney *U*‐test; *P < 0.05, **P < 0.01, ***P < 0.001.

Histological analysis of excised tumors using Ki67 immunohistochemistry revealed no significant differences in the number of labeled cells or in the tumor proliferation index (calculated as: [number of Ki67^+^ tumor cells/total number of tumor cells] × 100). These findings indicate that anti‐LVRF antibodies do not affect tumor cell proliferation (Fig. 6B–D). In contrast, CD31 immunohistochemistry revealed a significant reduction in the density of patent blood vessels in tumors treated with anti‐LVRF antibodies compared to controls (Fig. 6B–E). This was accompanied by a marked increase in the proportion of collapsed, nonfunctional CD31‐positive vessels (Fig. 6F), suggesting that LVRF blockade impairs vascular functionality and contributes to vascular normalization or collapse.

In parallel, analysis of the lymphatic compartment using LYVE‐1 immunohistochemistry demonstrated a substantial decrease in lymphatic vessel density in treated tumors (Fig. 6B–G). Moreover, most of the remaining lymphatic vessels displayed a collapsed morphology, with more than 70% of vessels affected in treated tumors compared to less than 20% in controls (*P* < 0.001; Fig. 6H).

Collectively, these findings demonstrate that anti‐LVRF antibodies exert a dual antivascular effect *in vivo* by disrupting both blood and lymphatic vessel integrity, while also directly inhibiting tumor growth. These results establish LVRF as a therapeutically relevant target in ccRCC and support further development of anti‐LVRF strategies for overcoming resistance to anti‐angiogenic therapies.

### The lead anti‐LVRF antibody P2H3 reduces distant metastasis in a zebrafish xenograft model

To specifically assess the antimetastatic potential of anti‐LVRF antibodies, we used a zebrafish xenograft metastasis model. Fluorescently labeled 786‐O cells were injected into the perivitelline region of zebrafish embryos, followed by treatment with either the P2H3 antibody or an isotype control for three days. Distant micro metastases were then quantified in secondary anatomical sites (Fig. 7A).

**Fig. 7:**
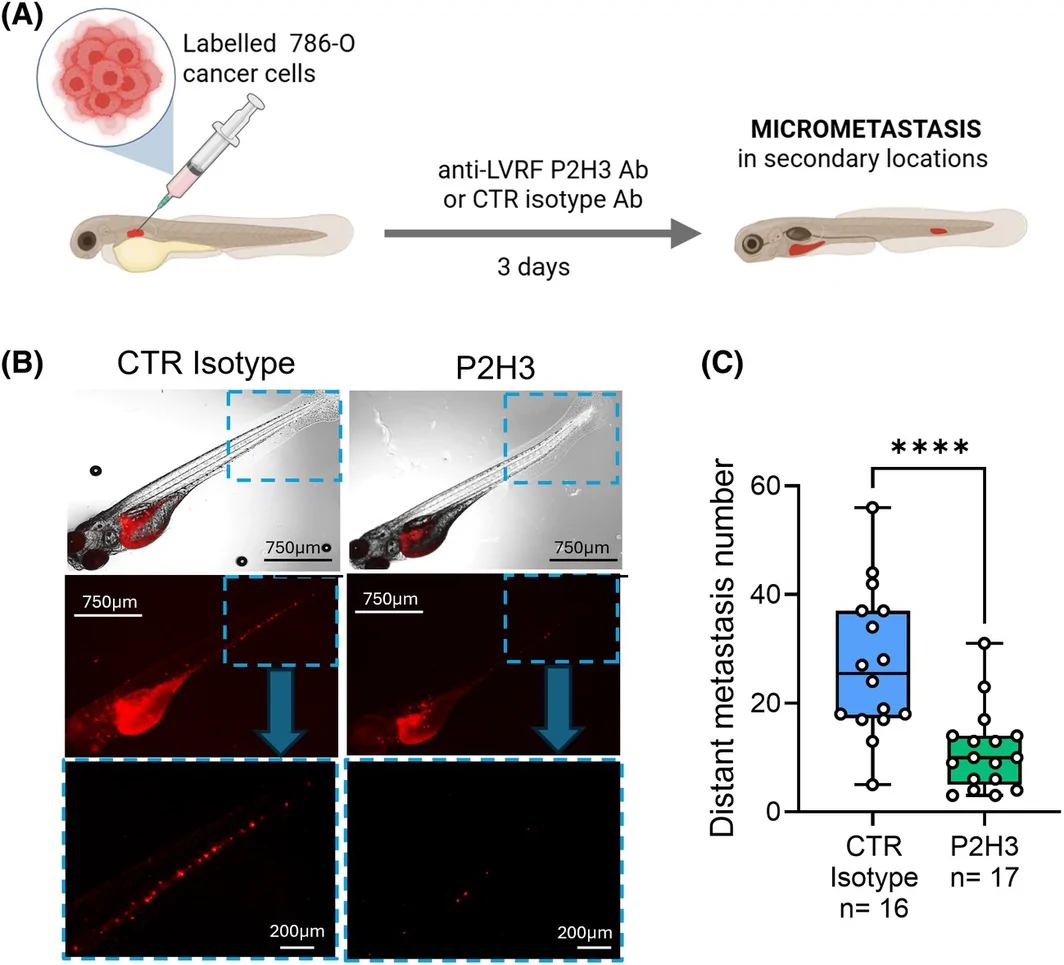
Anti‐LVRF monoclonal antibody P2H3 reduced distant metastasis in a zebrafish xenograft model. (A) Schematic representation of the zebrafish xenograft assay. Fluorescently labeled 786‐O cells were injected into the perivitelline cavity of zebrafish embryos, followed by treatment with P2H3 or isotype control antibody for 3 days. Created in BioRender. Pagès, G. (2026) https://BioRender.com/kdexkdk. (B) Representative images showing tumor cell dissemination in control and P2H3‐treated zebrafish. Scale bars, 750 μm in the upper and middle panels and 200 μm in the lower magnified panels. (C) Quantification of distant micrometastases. P2H3 reduced distant metastasis by 60%. Data are shown as box‐and‐whisker plots with individual data points. Boxes represent the interquartile range (25th–75th percentiles), the horizontal line indicates the median, and whiskers extend from the minimum to the maximum values. Statistical significance was assessed using the Mann–Whitney *U*‐test; ****P < 0.0001 (pooled from three independent experiments; isotype control, *n* = 16; P2H3, *n* = 17).

Treatment with P2H3 resulted in a significant 60% reduction in the number of distant metastatic foci compared to control antibody‐treated embryos (*P* < 0.0001, Mann–Whitney test; pooled from three independent experiments) (Fig. 7B,C). This robust and highly reproducible antimetastatic effect highlights the ability of LVRF neutralization to impair early dissemination and colonization steps of metastatic spread.

Importantly, this observation is consistent with the central role of LVRF in promoting lymphangiogenesis and vascular remodeling, both of which are key routes for tumor cell dissemination. These findings further reinforce the therapeutic relevance of targeting LVRF in highly metastatic cancers and support its potential as a strategy to limit systemic disease progression.

## Discussion

Anti‐angiogenic treatments, stemming from the pioneering work of J Folkman [1], have marked a significant stride in the management of various tumor types. The concept of angiogenesis is now firmly entrenched as one of the cancer hallmarks [2]. Nevertheless, the oversimplification of solely targeting VEGFA and its receptors to inhibit tumor angiogenesis disregards the complex dynamics at play during VEGFA or its receptor inhibition. Initially, anti‐angiogenic strategies were conceived under the premise that targeting normal cells, specifically endothelial cells, would evade the adaptive responses typically seen in cancer cells. However, many tumor cells also express VEGFA receptors or coreceptors, rendering them genetically responsive to this selection pressure.

Furthermore, VEGFA and its receptors/coreceptors are not limited to endothelial and tumor cells; immune cells, such as macrophages and lymphocytes, also express these molecules, influencing the adaptive immune response [27].

Since the discovery of VEGFA, several splice variants with distinct biological properties have been unveiled [3]. The existence of ‘anti‐angiogenic’ forms of VEGFA initially sparked debate, with some regarding them as artifacts. However, their presence in the plasma of metastatic colon cancer patients underscored the effectiveness of bevacizumab [21]. Contrarily, we have demonstrated the critical therapeutic value of specifically targeting the pro‐angiogenic VEGFA forms [32]. Our discovery of a novel splicing event has reshaped the VEGFA landscape, as altering the protein's C‐terminal region can convert it from a simple pro‐angiogenic factor to one with pro‐angiogenic and prolymphangiogenic properties [28]. While we continue to explore the full biology of this novel form, termed LVRF, we have emphasized its potential as a new therapeutic target. One intriguing characteristic is its relatively low abundance in the supernatant of normal cells but its elevation in tumor cells.

To expand therapeutic strategies for ccRCC, combining anti‐LVRF treatments with existing therapies holds significant potential. ccRCC remains a challenging disease due to its resistance to conventional therapies and the complexity of its angiogenic and immune microenvironment. Current standard‐of‐care treatments for advanced ccRCC include multitarget receptor tyrosine kinase inhibitors (RTKi), such as cabozantinib (targeting VEGFR, MET, and AXL) and lenvatinib (targeting VEGFR and fibroblast growth factor receptor/FGFR). These agents effectively inhibit both angiogenesis and tumor cell invasion, demonstrating clinical efficacy in overcoming resistance to first‐line anti‐VEGFA therapies [33, 34]. However, their long‐term benefits are often compromised by adaptive resistance mechanisms, including the upregulation of alternative pro‐angiogenic pathways, such as those mediated by LVRF. A combination strategy incorporating anti‐LVRF antibodies with cabozantinib or lenvatinib could therefore offer a synergistic therapeutic approach. By concurrently blocking VEGFR‐dependent signaling and LVRF‐mediated resistance pathways, such combinations may enhance antitumor efficacy and delay the onset of resistance.

Another cornerstone of ccRCC treatment is immune checkpoint inhibitors (ICIs), exemplified by nivolumab (anti‐PD‐1) and ipilimumab (anti‐CTLA‐4), which restore T‐cell‐mediated antitumor immunity [35]. The interplay between angiogenesis and immune suppression in ccRCC is well‐documented, with VEGFA and other angiogenic factors contributing to an immunosuppressive tumor microenvironment. By targeting LVRF, which promotes both angiogenesis and lymphangiogenesis, anti‐LVRF therapy could enhance the efficacy of ICIs by normalizing tumor vasculature and reducing immune exclusion. This rationale is supported by preclinical and clinical evidence demonstrating that anti‐angiogenic therapies improve immune cell infiltration and response to ICIs.

Currently, the combination of ICI and RTKIs represents the standard of care for patients with good and intermediate prognoses. Notable examples include axitinib plus pembrolizumab (anti‐PD‐1) [36], cabozantinib plus nivolumab [37], or lenvatinib plus pembrolizumab [38]. A triple combination incorporating anti‐LVRF could represent an innovative and promising approach to further enhance therapeutic outcomes.

Finally, the recent approval of belzutifan, a first‐in‐class HIF‐2α inhibitor, marks a significant advancement in targeting the hypoxia‐driven biology of ccRCC [39]. While the combination of belzutifan with pembrolizumab has shown some toxic effects, it significantly increases progression‐free survival compared to pembrolizumab alone [40]. Given that LVRF expression is influenced by HIF pathways, though not exclusively, combining anti‐LVRF with belzutifan could offer a complementary strategy to disrupt both HIF‐dependent and HIF‐independent angiogenic resistance mechanisms. Such a combination may be particularly effective in tumors where LVRF is upregulated as a compensatory response to HIF‐2α inhibition.

As previously discussed, we conducted a pivotal translational study, revealing the presence of LVRF in the plasma of ccRCC patients. The correlation between high LVRF levels and progression‐free survival after sunitinib treatment, as well as overall survival, marked a significant breakthrough. Importantly, the range of LVRF levels in plasma was vast, ranging from undetectable to as high as 6 ng·mL^−1^ [28].

These results reveal that not all patients may benefit from treatments targeting LVRF. However, this study focuses on defining the target product profile for specific antibodies against LVRF. Patient‐derived xenograft (PDX) screening played a crucial role in this, as we detected LVRF positivity in low numbers in several tumor types. Notably, colon and ovarian cancers [12, 15] and ccRCC [11] where bevacizumab is a standard of care, are promising candidates for assessment, as are medulloblastomas, and ependymoma which showed significant benefits in recent clinical trials [41, 42, 43, 44]. Given the ubiquity of resistance to anti‐angiogenic drugs, LVRF's presence in various tumors suggests a potential mechanism of resistance following treatment or at the diagnosis of metastatic disease. Other PDX showed the presence of LVRF and could therefore represent interesting tumors to target.

The selection of our lead antibody candidate, P2H3, was guided by a comprehensive evaluation that extended beyond efficacy to include critical developability, stability, and manufacturability criteria. While four monoclonal antibodies (P2H3, P1F12, P1C9, and P1B7) demonstrated comparable affinities and biological activities in initial screening, P2H3 emerged as the optimal candidate for clinical translation. This decision was based on its superior stability, favorable pharmacokinetic profile, and lower immunogenicity risk, all of which are essential for scalable production and long‐term therapeutic use. Although P1F12 exhibited strong antitumor activity in preclinical models, P2H3 was prioritized due to its balanced profile, including robust efficacy in both *in vitro* and *in vivo* assays, as well as its compatibility with large‐scale manufacturing processes. This multifactorial approach ensures that P2H3 not only effectively targets LVRF but also meets the rigorous demands of clinical development, positioning it as a promising therapeutic agent for further investigation.

In this study, we describe three human antibodies that displayed robust antitumor responses, with tumor growth inhibition rates of 58−64%, surpassing axitinib, a standard of care. These antibodies exhibited their potent inhibitory potential even in a model insensitive to bevacizumab. Moreover, beyond curtailing tumor growth, they also demonstrated a marked reduction in tumor neovascularization.

Anti‐LVRF therapy is positioned as a targeted intervention for patients with highly vascularized and metastatic cancers, particularly those that develop resistance to standard anti‐angiogenic therapies. Given the role of LVRF in promoting both angiogenesis and lymphangiogenesis, its targeting is most relevant in patients who have progressed on first‐line anti‐VEGFA treatments, such as bevacizumab or sunitinib. In this context, anti‐LVRF therapy could serve as a second‐line or later treatment option for anti‐VEGFA‐resistant tumors, where LVRF upregulation is a known mechanism of resistance. Additionally, anti‐LVRF antibodies could be combined with existing therapies, such as RTKIs cabozantinib/lenvatinib/axitinib, ICIs like nivolumab or ipilimumab, or HIF2α inhibitors like belzutifan, to enhance efficacy and overcome resistance mechanisms. Given LVRF's role in promoting tumor invasion and metastatic dissemination, anti‐LVRF therapy may be particularly beneficial for patients with advanced or metastatic disease, where lymphangiogenesis and angiogenesis contribute to poor prognosis.

To maximize the clinical benefit of anti‐LVRF therapy, patient selection should be guided by biomarker‐driven approaches. Tissue‐based LVRF expression can be assessed directly using immunohistochemistry or RNA *in situ* hybridization, which provide spatial context and enable correlation with tumor vascularization and metastatic potential. Next‐generation sequencing or RT‐qPCR can also quantify LVRF mRNA levels in tumor biopsies, offering a precise measurement of LVRF upregulation. Circulating LVRF levels in plasma could serve as a noninvasive biomarker for patient selection and monitoring. Given that LVRF is induced by anti‐angiogenic therapy, its detection in circulation could identify patients who are developing resistance and may benefit from anti‐LVRF treatment. Dynamic monitoring through serial measurements of circulating LVRF during anti‐VEGFA therapy could help predict the onset of resistance and guide the timing of anti‐LVRF intervention. A dual approach, integrating both tissue and circulating LVRF assessments, may provide the most robust strategy for patient selection. For example, baseline LVRF expression in tumor tissue could identify patients with intrinsic LVRF‐driven angiogenesis, while an increase in circulating LVRF during treatment could signal the emergence of resistance, prompting the initiation of anti‐LVRF therapy.

While the translational potential of anti‐LVRF therapy is promising, several challenges need to be addressed. The heterogeneity of LVRF expression across tumor types and within individual tumors underscores the need for precise biomarker stratification to identify the most responsive patient populations. Although LVRF is expressed at low levels in some normal tissues, its tumor‐specific upregulation and functional role in resistance suggest a therapeutic window that can be exploited with careful dosing and monitoring. The optimal sequencing and dosing of anti‐LVRF therapy in combination with other agents, such as RTKis, ICIs, or HIF2α inhibitors, will require further preclinical and clinical investigation to maximize efficacy and minimize toxicity. To advance clinical development of anti‐LVRF therapy, we propose the following steps. Prospective studies should validate LVRF as a predictive biomarker by correlating its expression in tissue and circulation with response to anti‐LVRF therapy in preclinical models and early‐phase clinical trials. The development of companion diagnostics, such as standardized assays for LVRF detection in tissue and circulation, will facilitate patient selection in clinical practice. Preclinical studies should also explore the efficacy of anti‐LVRF antibodies in combination with existing therapies to identify synergistic effects and optimize treatment regimens.

We firmly believe that these anti‐LVRF antibodies could set a new standard of care and merit testing in future basket clinical trials, enrolling patients with metastatic ccRCC, colon, and ovarian cancers that have relapsed on conventional treatments. Furthermore, given the well‐established immunoregulatory functions of VEGFA [27] and the potential role of LVRF in modulating T‐lymphocyte activity through the induction of immune checkpoint inhibitor expression, anti‐LVRF antibodies may represent a promising strategy to restore antitumor immune responses. By counteracting these immunosuppressive mechanisms, LVRF targeting could contribute to immune reactivation and thereby exert a substantial therapeutic benefit, particularly in tumors characterized by an immunosuppressive microenvironment.

This study demonstrates the promising therapeutic potential of targeting LVRF in highly vascularized and metastatic cancers, particularly in overcoming resistance to anti‐angiogenic therapies. However, some limitations should be noted. The preclinical models used, including xenografts in immunodeficient mice and zebrafish embryos, provide valuable insights but may not fully replicate the complexity of human tumor microenvironments, including immune interactions. While LVRF expression was observed across multiple tumor types, its variable levels suggest that patient selection based on biomarker stratification will be important for optimizing clinical outcomes. Additionally, the focus on the 786‐O cell line and bevacizumab‐resistant models provides a strong foundation, but further validation in diverse tumor models will strengthen the generalizability of these findings. Finally, while the anti‐LVRF antibodies exhibited high specificity and efficacy, long‐term safety and potential effects on normal tissues expressing low LVRF levels will require additional investigation. These limitations are outweighed by the study's robust preclinical evidence, which supports the advancement of anti‐LVRF therapies into clinical evaluation, where their full potential can be further explored.

## Conclusion

This study identifies LVRF, a VEGFA splice variant, as a key driver of angiogenesis, lymphangiogenesis, and resistance to anti‐angiogenic therapy in renal cell carcinoma. We show that fully human anti‐LVRF antibodies effectively block tumor vascularization, invasion, and metastasis, including in bevacizumab‐resistant models. These findings establish LVRF as a clinically relevant therapeutic target and support anti‐LVRF antibodies as a promising strategy to overcome resistance to current anti‐VEGFA treatments.

## Conflict of interest

GP, ZS, and OL hold shares in Kekkan Biologics. ZS, OL and MD are co‐owners of a patent held by Kekkan Biologics. MD is an employee of Kekkan Biologics.

## Author contributions

GP and MD conceived and supervised the study; GP, MD, AK, and OL designed experiments; AK, AB, CF, QO, AG, IB, CG, SG, and FL performed experiments; MD and AK analyzed data; GP and MD wrote the manuscript; GP and ZS acquired funding. All authors have read and agreed to the published version of the manuscript.

## Supporting information


**Fig. S1.** Phage display generation of anti‐LVRF antibodies. A human single‐chain fragment variable (scFv) phage display library (human LiAb‐SFCANCER™ library in mix with human LiAb‐ 1.46 × 10^11^ variants) was screened against the LVRF‐specific C‐terminal peptide (DVTSRGGEPGRRKEPPSGFREPDLS) over three rounds of bio panning with alternating carrier conjugates (OVA, BSA, KLH) and carrier pre‐depletion. The round 3 output (7.8 × 10⁷ pfu) was used for monoclonal phage ELISA screening of 288 single clones across four 96‐well plates, yielding 47 positive clones that were sequenced. The 12 clones recognizing full‐length LVRF and exhibiting diverse sequences were selected for recombinant IgG1 production.
**Fig. S2.** Anti‐LVRF antibodies inhibit lymphangiogenesis. Representative images of lymphatic endothelial cell (LEC) sprouting in a 3D bead assay under control conditions, stimulation with recombinant LVRF (200 ng·mL^−1^), or LVRF in the presence of anti‐LVRF antibodies (100 μg·mL^−1^) (*n* = 3). Scale bars, 100 μm.

## Data Availability

The data underlying this article are available in the article and in its online Supporting Informations. Further inquiries can be directed at the corresponding author.
