# Supplementary material for: Characterization of therapeutic antibodies targeting lymphatic and vascular resistance factor for the treatment of hypervascularized and highly metastatic cancers
Source: Mol Oncol. 2026 Oct 20:10.1002/1878-0261.70343. Online ahead of print. doi: 10.1002/1878-0261.70343 (PMC13682602; doi:10.1002/1878-0261.70343)
Supplement: Supplementary file 1 [file MOL2-9999-0-s001.docx]

**SUPPLEMENTARY FIGURE :**

**Figure S1.** Phage display generation of anti-LVRF antibodies.

A human single-chain fragment variable (scFv) phage display library (human LiAb-SFCANCER™ library in mix with human LiAb- 1.46 × 10¹¹ variants) was screened against the LVRF-specific C-terminal peptide (DVTSRGGEPGRRKEPPSGFREPDLS) over three rounds of bio panning with alternating carrier conjugates (OVA, BSA, KLH) and carrier pre-depletion. The round 3 output (7.8 × 10⁷ pfu) was used for monoclonal phage ELISA screening of 288 single clones across four 96-well plates, yielding 47 positive clones that were sequenced. The 12 clones recognizing full-length LVRF and exhibiting diverse sequences were selected for recombinant IgG1 production.

**Figure S2**: Anti-LVRF antibodies inhibit lymphangiogenesis.

Representative images of lymphatic endothelial cell (LEC) sprouting in a 3D bead assay under control conditions, stimulation with recombinant LVRF (200 ng/mL), or LVRF in the presence of anti-LVRF antibodies (100 µg/mL) (n=3). Scale bars, 100 µm
